# Radiomics features of DSC-PWI in time dimension may provide a new chance to identify ischemic stroke

**DOI:** 10.3389/fneur.2022.889090

**Published:** 2022-11-04

**Authors:** Yingwei Guo, Yingjian Yang, Fengqiu Cao, Yang Liu, Wei Li, Chaoran Yang, Mengting Feng, Yu Luo, Lei Cheng, Qiang Li, Xueqiang Zeng, Xiaoqiang Miao, Longyu Li, Weiyan Qiu, Yan Kang

**Affiliations:** ^1^College of Medicine and Biological Information Engineering, Northeastern University, Shenyang, China; ^2^Medical Device Innovation Center, Shenzhen Technology University, Shenzhen, China; ^3^Department of Radiology, Shanghai Fourth People's Hospital Affiliated to Tongji University School of Medicine, Shanghai, China; ^4^Shenzhen Happy-Growing Intelligent CO., Ltd, Shenzhen, China; ^5^School of Biomedical Engineering, Shanghai Jiao Tong University, Shanghai, China; ^6^Engineering Research Centre of Medical Imaging and Intelligent Analysis, Ministry of Education, Shenyang, China

**Keywords:** ischemic stroke, hypoperfusion area, radiomics, feature selection, DSC-PWI

## Abstract

Ischemic stroke has become a severe disease endangering human life. However, few studies have analyzed the radiomics features that are of great clinical significance for the diagnosis, treatment, and prognosis of patients with ischemic stroke. Due to sufficient cerebral blood flow information in dynamic susceptibility contrast perfusion-weighted imaging (DSC-PWI) images, this study aims to find the critical features hidden in DSC-PWI images to characterize hypoperfusion areas (HA) and normal areas (NA). This study retrospectively analyzed 80 DSC-PWI data of 56 patients with ischemic stroke from 2013 to 2016. For exploring features in HA and NA,13 feature sets (*F*_*method*_) were obtained from different feature selection algorithms. Furthermore, these 13 *F*_*method*_ were validated in identifying HA and NA and distinguishing the proportion of ischemic lesions in brain tissue. In identifying HA and NA, the composite score (CS) of the 13 *F*_*method*_ ranged from 0.624 to 0.925. *F*_*Lasso*_ in the 13 *F*_*method*_ achieved the best performance with mAcc of 0.958, mPre of 0.96, mAuc of 0.982, mF1 of 0.959, and mRecall of 0.96. As to classifying the proportion of the ischemic region, the best CS was 0.786, with Acc of 0.888 and Pre of 0.863. The classification ability was relatively stable when the reference threshold (*RT*) was <0.25. Otherwise, when *RT* was >0.25, the performance will gradually decrease as its increases. These results showed that radiomics features extracted from the Lasso algorithms could accurately reflect cerebral blood flow changes and classify HA and NA. Besides, In the event of ischemic stroke, the ability of radiomics features to distinguish the proportion of ischemic areas needs to be improved. Further research should be conducted on feature engineering, model optimization, and the universality of the algorithms in the future.

## Introduction

Ischemic stroke is a significant cause of death worldwide and has a heavy toll on death and disability (1). Therefore, the warning symptoms, clinical features, and prognostic evaluation around stroke have always been the subject of clinical and scientific research. Research shows that the initiating presentation of ischemic stroke is the occlusion of a blood vessel that impairs blood flow to a certain degree, leading to infarction of brain tissue in the part of the brain supplied by that blood vessel (2, 3). That means the state of cerebral blood flow has become a significant factor for the early warning and status assessment of stroke, and the early detection of abnormal blood flow is of great significance for timely treatment and excellent prognosis of patients.

To identify the presence of reduced regional blood flow, studies and physicians have combined diverse modalities of images with various analysis methods to detect abnormal states and identify hypoperfusion areas (HA) that may cause a stroke. In most imaging modes, perfusion images, such as dynamic susceptibility contrast perfusion-weighted imaging (DSC-PWI) and computed tomography perfusion (CTP), play a vital role in stroke analytics in clinical practice and trials due to their ability to evaluate cerebral blood flow state. When the contrast agent arrives at the ill-perfused tissue of the brain, the signal intensity values barely change since there is no or less propagation of the contrast agent to the damaged tissue (4). Thus, the time to the maximum tissue residual function (*T*_*max*_) obtained from DSC-PWI, a highly commonly used parameter, has been used in clinical trials to identify the HA (5, 6). Generally, the region recognized from the condition *T*_*max*_ >6s is defined as HA (7). In addition, other single-modality images except for DSC-PWI, or in combination with it, can also provide much medical information on ischemic stroke. A study (8) shows that the mismatch between DSC-PWI and diffusion-weighted imaging (DWI) has been used to estimate the ischemic penumbra and provides a valuable tool in the clinical treatment of stroke, which helps guide the selection of the clinical therapeutic plan. Lu et al. (9) evaluated the volume of the ischemic penumbra using susceptibility-weighted imaging and mapping (SWIM) of patients with asymmetrical prominent cortical veins. Wang et al. (10) discussed the value of susceptibility-weighted imaging (SWI) in evaluating the ischemic penumbra of patients with acute cerebral ischemic stroke. Bhattacharjee et al. (11) verified that the quantitative assessment of the penumbra using the SWI-DWI mismatch ratio performs equivalently to the ASL, PWI-DWI mismatch ratio. Furthermore, continuously developed artificial intelligence models can interpret and analyze the manifestations of stroke (12–14). Although many previous studies have been committed to evaluating the HA from multimodal imaging manners, multidimensional analysis methods, and advanced artificial intelligence technology, there are few methods to analyze the image features themselves to discover the association between the image features and cerebral blood flow state.

Radiomics is an emerging methodology that quantifies high-dimensional features from imaging data and has been used to investigate tumor heterogeneity (15, 16) and for clinical decision support systems to improve treatment decision-making and accelerate advancements toward precision medicine in cancer (14, 17–21). Recently, only a tiny minority of studies have investigated the role of radiomics in identifying ischemic stroke lesions (22), evaluating prognostic biomarkers based on the penumbra (23), and predicting functional outcomes (24). However, these studies combined medical images with clinical text information to perform the above tasks but ignored the features themselves. Currently, few studies have explored the association between imaging characteristics in the temporal dimension of DSC-PWI and blood flow status in ischemic stroke. However, with abundant and distinct blood flow information in DSC-PWI data, it is possible to extract these features to explain the blood flow state.

As for classification tasks, machine learning models and neural networks have been widely used for a long time. However, each method has its rules and algorithms to perform tasks. For example, Logistic Regression (LR) (25–27) quantifies the coefficients of variables to predict a logit transformation of the probability of the presence of the event. Support Vector Machine (SVM) (28) learns an optimal hyperplane that separates the classes as widely as possible. SVM can also perform nonlinear classification using the “kernel” to map to higher dimensional feature space (29). Random Forest (RF) is created based on decision trees (DT). Their methods resemble human reasoning by representing hypotheses as sequential if-then. The AdaBoost algorithm (Ada) (30) corrects the misclassifications made by weak classifiers, and it is less susceptible to overfitting than most learning algorithms. Gradient Boosting Decision Tree (GBDT) adapts the boosting algorithm, and it uses the error rate of the previous iteration weak learner to update the weight of the training set (31). Besides, the k-nearest neighbor (KNN) is a non-parametric classification method that forms the k neighborhood for features (32). The Naive Bayes classifier (NB) is a simple probabilistic classifier based on Bayes's theorem under solid independence between components (33). In addition to numerous machine learning models, neural networks, such as Multilayer Perceptrons (MLP) and Convolutional Neural Networks (CNN) (34–36), are commonly used to perform classification tasks. Generally, a single model is usually selected for task execution in the current classification tasks. However, as there are more or fewer differences between the algorithms of different models, the comprehensive evaluation of the results through multiple models will increase credibility. Thus, if we can verify the performance of the image features of DSC-PWI data in identifying ischemic stroke through models with different preferences, it will undoubtedly improve the validation accuracy and enhance the persuasiveness.

The purpose of this paper mainly consists of two aspects. The first is to discover the image features hidden in DSC-PWI data that can accurately distinguish normal tissues from abnormal tissues. The second is to explore the changes in the classification task when the proportion of abnormal tissues is different.

## Materials and methods

Detailed materials and methods are introduced in the following. The procedures in this study include making HA and normal area (NA), computing radiomics features, selecting excellent features, and evaluating radiomics feature sets.

### Materials

This retrospective study was approved by the Institutional Review Boards of Shanghai Fourth People's Hospital Affiliated with Tongji University School of Medicine and exempted from informed consent. The datasets in our study were collected by the neurology department of Shanghai Fourth People's Hospital Affiliated to Tongji University School of Medicine, China, from 2013 to 2016. In total, 80 DSC-PWI images of 56 patients with ischemic stroke were retrospectively reviewed and included. All patients were imaged within 24 h of symptom onset, and 22 patients were screened at least twice during pre and post treatment. Of all the patients, 26 patients presented with ischemic lesions in the left hemisphere, 28 in the right, and 26 in both. At least two experienced clinicians determined these diagnoses. The DSC-PWI image for each patient was scanned on a 1.5T MR scanner (Siemens, Germany), and Table 1 shows the details.

**Table 1:** Summary of patient information and the scanning parameters of DSC-PWI images.

**Information of patients**		**Scanning parameters of DSC-PWI images**	
Numbers of patients	56	TE/TR	32 ms/1,590 ms
Datasets (sets)	80	Matrix	256 × 256
Number of female patients (%)	15 (26.79%)	FOV	230 × 230 mm^2^
Age (Mean ± Std)	71 ± 11	Thickness	5 mm
HA in Left (%)	26 (32.5%)	Number of measurements	50
HA in right (%)	28 (35%)	Spacing between slices	6.5 mm
HA in both (%)	26 (32.5%)	Pixel bandwidth	1,347 Hz/pixel
Volume of HA (Mean ± Std, ml)	95.58 ± 75.23	Number of slices	20
NHISS (Mean ± Std)	9.225 ± 7.135		

### Methods

**Figure 1:**
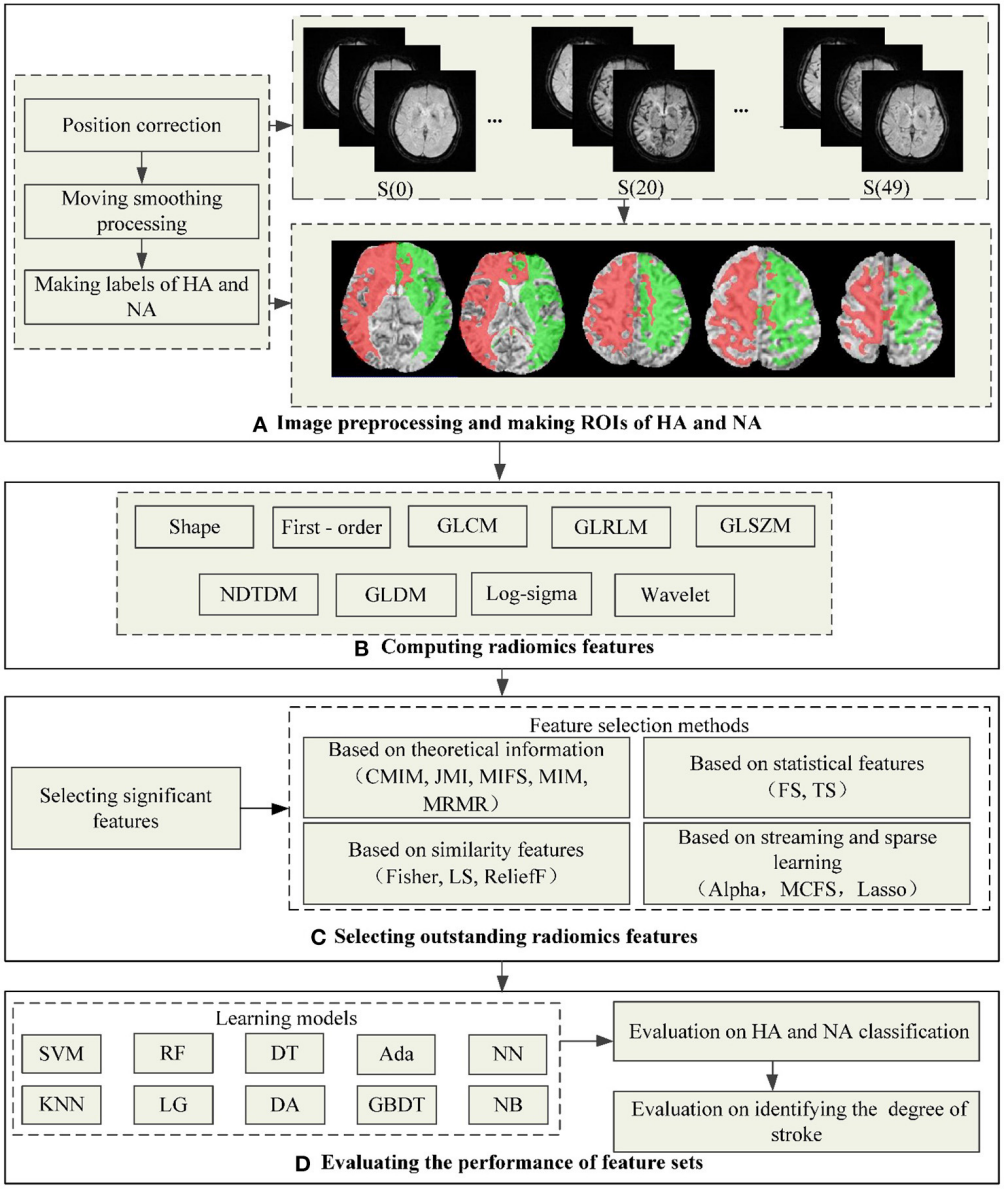
The flowchart of the proposed method in this study. **(A)** Shows the process of preprocessing images and making ROIs of HA and NA, wherein the red area is HA and the green is NA. **(B–D)** Show the process of computing radiomics features, outstanding feature selection, and evaluating the performance of feature sets.

Figure 1 shows the flowchart of the proposed method in this study, including preprocessing datasets and making ROIs, computing radiomics features, selecting outstanding features, and evaluating radiomics feature sets. The following is a detailed description of the process.

#### Preprocessing DSC-PWI images and making regions of interest (ROIs)

Preprocessing the datasets is intended to reduce noise and position deviation impacts. Firstly, we corrected DSC-PWI datasets for potential patient motion by registering all the volumes in the time series with the multiplicative intrinsic component optimization algorithm (37, 38). Then, we performed a data smoothing filtering to decrease the noise interference while preserving signal accuracy. In detail, the triple moving average filter was selected to smooth the data voxel-by-voxel with a 1 × 3 filtering kernel. In the DSC-PWI data, the intensity of each pixel in the time dimension forms a time-intensity sequence I(t) with noise generated from the equipment and other factors, while the smoothed I(t) decreased this trouble.

In addition, the necessary condition for comparative analysis of HA and NA is to detect both locations accurately. In this study, we used a fully automated Rapid Processing of Perfusion and Diffusion (RAPID) software (iSchemaView, CA, USA) (39) to segment the HA in the brain, and the segment condition was *T*_*max*_ > 6 s. In contrast with HA, we determined the healthy area in the symmetrical region of HA as NA. Therefore, 80 ROIs for HA and NA were generated from the DSC-PWI datasets.

#### Calculating radiomics features

Radiomics refers to the high-throughput extraction and analysis of many advanced and quantitative imaging features from medical images such as computed tomography (CT), positron emission computed tomography (PET), or magnetic resonance imaging (MRI). This study innovatively applied the Radiomics technology to DSC-PWI images to obtain the image features on the time dimension in each NA and HA. In detail, the DSC-PWI datasets are the four-dimensional (4D) images composed of N three-dimensional (3D) images with the size of S×H×W. Wherein N is the total number of the 3D images in the time dimension, and S, H, and W represent the slice numbers, height, and width of the 3D image, respectively. By decomposing the 4D data into N (50 in this study) single 3D images, the radiomics features for each 3D image can be computed separately. Then, a total of 65,800 radiomics features (50 3D images ×1316 features) can be calculated from each DSC-PWI data. These radiomics features were divided into nine groups: (1) Shape-based (Shape, 14 features × 50 = 700 features), (2) First Order Statistics (Firs*t-*order, 18 features × 50 = 900 features), (3) Gray Level Co-occurrence Matrix (GLCM, 24 features × 50 = 1,200 features), (4) Gray Level Run Length Matrix (GLRLM, 16 features × 50 = 800 features), (5) Gray Level Size Zone Matrix (GLSZM, 16 features × 50 = 800 features), (6) Neighboring Gray Tone Difference Matrix (NGTDM, 5 features×50 = 250 features), (7) Gray Level Dependence Matrix (GLDM, 14 features × 50 = 700 features), (8) Log-sigma (Log-sigma, 465 features× 50 = 23250 features), (9) Wavele*t-*based (Wavelet, 744 features × 50 = 37,200 features). Feature calculations were automatically performed using the PyRadiomics package implemented in Python (40, 41). In this study, the definition of each radiomics feature was combined with the name of the radiomics feature itself and the time value of the 3D image connected by the underline, wherein n is the time value corresponding to the 3D image, *n* ∈ [0,49]. Each 3D image in DSC-PWI data can be defined as S(n), where n is the time value of the 3D image and ranges from 0 to 49. For example, “log-sigma-1-0-mm-3D_firstorder_Skewness_17” represents the radiomics feature “log-sigma-1-0-mm-3D_firstorder_Skewness” of S(3), which is the fourth 3D image in DSC-PWI data, and the feature belongs to the Log-sigma group. In this study, the *p-*value of each radiomics feature was obtained from the *T-*test operation, and their statistics (mean, std, minimum, median, and maximum) can be calculated by the Origin 2021 software.

#### Selecting outstanding radiomics features

##### Selecting significant features

*T-*test analysis was performed to reduce the feature dimensionality while retaining significant features to the greatest extent. By the *T-*test analysis, the significant features between NA and HA can be extracted. Before the *T-*test analysis, a normalization operation was performed according to Equation (1). Finally, 19857 significant features with *p-*values lower than 0.05 remained to complete subsequent feature selection processing.


(1) $$\begin{array}{l} F_{i}^{*}=\left(F_{i}-\bar{F_{i}}\right)/\left(F_{imax}-F_{imin}\right) \end{array}$$

Wherein *F*_*i*_ is the i*th* feature in all the 65,800 radiomics features, the variables *F*_*i*_, *F*_*imax*_, and *F*_*imin*_ are the mean, maximum, and minimum of *F*_*i*_, respectively.

##### Selecting multiple feature sets from diverse methods

One purpose of feature selection was to find the most compelling feature representing the target variable; the other was to compress feature space. This study used multiple feature selection methods based on diversity principles to select outstanding features from the 19,857 significant features. The feature selection methods contained four types: the methods based on theoretical Information [FI, including Conditional Mutual Information Maximization (CMIM), Joint Mutual Information (JMI), Mutual Information Feature Selection (MIFS), Mutual Information Maximization (MIM), and Minimal Redundancy Maximum Relevance (MRMR)], based on similarity features [SIF, including Fisher-score (Fisher), Lap-score (Lap), and (ReliefF)], based on the statistical features [STF, including F-score (FS), *T-*score (TS)], and based on sparse learning and steaming [SSL, including multi-cluster feature selection (MCFS), Alpha-investing (Alpha), the least absolute shrinkage and selection operator (Lasso)]. The above methods were introduced in reference (42–47), described in Table 2, and implemented in Python 3.6.

**Table 2:** Descriptions of the 13 feature selection methods used in this study.

**Type**	**Method**	**Description**	**Equation**
FI	MIM	Evaluating features by the correlation between features and classes measured by the mutual information	*MIM*(*f*_*i*_) = *I*(*f*_*i*_; *C*)
	MIFS/MRMR	Evaluating features by the correlation between features and classes, and redundancy among features	$MIFS\left(f_{i}\right)=I\left(f_{i};C\right)-\beta \underset{s_{j}\in S}{\sum }I\left(f_{i};f_{S}\right)$ $MRMR\left(f_{i}\right)=I\left(f_{i};C\right)-\frac{1}{S}\underset{s_{j}\in S}{\sum }I\left(f_{i};f_{S}\right)$
	JMI/CMIM	Evaluating features by the correlation between features and classes, and redundancy among features measured by the conditional mutual information	$JMI\left(f_{i}\right)=I\left(f_{i};C\right)-\frac{1}{\left|S\right|}\underset{s_{j}\in S}{\sum }\left[I\left(f_{i};C\right)-I\left(f_{i};C|f_{S}\right)\right]$ $CMIM\left(f_{i}\right)=\underset{f_{s}\in S}{\min}I\left(f_{i};C|f_{s}\right)$
SIF	Fisher/Lap	Comparing features with their ratios of the variance between classes and the variance within classes	$\text{Fisher}\left(k\right)=\frac{R_{B}^{\left(k\right)}}{R_{w}^{\left(k\right)}}$ $LS\left(f_{i}\right)=\frac{\underset{ab}{\sum }{\left(f_{ra}-f_{rb}\right)}^{2}W_{ij}}{Var\left(f_{r}\right)}$
	ReliefF	Comparing features with the correlation between features and classes computed from the ability of features to distinguish between close samples	$ReliefF\left(f_{i},R1,R2\right)=\frac{\left|R1\left(A\right)-R2\left(A\right)\right|}{\max\left(A\right)-\min\left(A\right)}$
STF	FS	obtaining feature score with the ability to distinguish positive class and negative class computed by the average of both classes	$FS\left(i\right)=\frac{{\left({{\bar{f}}_{i}}^{\left(+\right)}-{\bar{f}}_{i}\right)}^{2}+{\left({{\bar{f}}_{i}}^{\left(-\right)}-{\bar{f}}_{i}\right)}^{2}}{\frac{1}{n_{+}-1}\overset{n_{+}}{\underset{k=1}{\sum }}{\left({f_{k,i}}^{\left(+\right)}-{{\bar{f}}_{i}}^{\left(+\right)}\right)}^{2}+\frac{1}{n_{-}-1}\overset{n_{-}}{\underset{k=1}{\sum }}{\left({f_{k,i}}^{\left(-\right)}-{{\bar{f}}_{i}}^{\left(-\right)}\right)}^{2}}$
	TS	Computing feature score with the average and variance of features	$TS\left(i\right)=\frac{\left({{\bar{f}}_{i}}^{\left(+\right)}-{{\bar{f}}_{i}}^{\left(-\right)}\right)}{\frac{1}{n_{+}-1}\overset{n_{+}}{\underset{k=1}{\sum }}{\left({f_{k,i}}^{\left(+\right)}-{{\bar{f}}_{i}}^{\left(+\right)}\right)}^{2}+\frac{1}{n_{-}-1}\overset{n_{-}}{\underset{k=1}{\sum }}{\left({f_{k,i}}^{\left(-\right)}-{{\bar{f}}_{i}}^{\left(-\right)}\right)}^{2}}$
SSL	MCFS	Combing cluster with feature coefficients of combinatorial classes to compute feature score	$MCFS\left(i\right)=\frac{\max}{k}|f_{k,i}|$
	Alpha	Evaluating features by dynamically adjusting the threshold on the error reduction to obtain selection results	*E*(*N*_*i*_)/*E*(*M*_*i*_) < α_Δ_/(1−α_Δ_)
	Lasso	Using L1 regularization to make the weight of some learned features 0, to achieve the purpose of sparse and feature selection	$Lasso\left(\overset{\wedge }{\beta }\right)=arg\;min\left\{\overset{n}{\underset{i=1}{\sum }}{\left(y_{\text{i}}-\beta_{0}-\overset{p}{\underset{j=1}{\sum }}\beta_{j}x_{ij}^{*}\right)}^{2}+\lambda \overset{p}{\underset{j=0}{\sum }}|\beta_{j}|\right\}$

During the implementation of each method, except that Lasso selects features with coefficients more prominent than 0.02 to control the number of features within the set max feature-length 20, the others obtained the features whose score exceeded 0.9 and the total number was <20. To distinguish between features obtained from the 13 method, the features extracted from them were regarded as feature set *F*_*method*_, wherein the “*method”* represents the name of the technique (CMIM, JMI, MIFS, MIM, MRMR, Fisher, Lap, ReliefF, MCFS, Alpha, Lasso, FS, TS). Besides, the features obtained from techniques in the same type were regarded as *F*_*type*_, the “*type*” was the category to which the method belongs, type∈{FI, SIF, STF, SSL}, and all the selected features were named *F*_*all*_.

#### Performance evaluation of the selected features

This study evaluated the feature sets in two aspects, including the performance of identifying HA from NA on multiple models and the classification ability to determine the proportion of stroke regions in the brain hemispheres.

##### Evaluating the performance of classifying HA from NA

We applied ten commonly used supervised machine learning models to identify HA and NA by learning each feature set *F*_*method*_. The machine learning models included support vector machines (SVM), decision tree (DT), Adaboost classifier (Ada), neural network (NN), random forest (RF), k nearest neighbors (KNN), logistic regression (LR), linear discriminant analysis (DA), gradient boosting classifier (GBDT), and GaussianNB (NB) (seen in Table 3). By training the ten models with the 13 *F*_*method*_, 130 (13 × 10) classifiers were created. These classifiers were defined by combining the learning machine model and the feature selection method. For example, the *C*_*SVM*_*MIM*_ represents the classifier fitted by SVM and feature sets *F*_*MIM*_, while *C*_*SVM*_*SIF*_ means the classifier generated from SVM and all the feature sets *F*_*SIF*_.

**Table 3:** Descriptions of 10 models in this study.

**No**.	**Model**	**Definition in python 3.6**
1	SVM	sklearn.svm.SVC(kernel=‘rbf',probability=True)
2	DT	sklearn.tree. DecisionTreeClassifier()
3	Ada	sklearn.ensemble.AdaBoostClassifier()
4	NN	sklearn.neural_network. MLPClassifier (hidden_layer_size*S =* (400, 100), alpha=0.01, max_iter=10000)
5	RF	sklearn.ensemble.RandomForestClassifier(n_estimator*S =* 200)
6	KNN	sklearn.neighbors. sklearn.neighbors()
7	LR	sklearn.linear_model.logisticRegressionCV(max_iter=100000, solver=“liblinear”)
8	DA	sklearn.discriminant_analysis.()
9	GBDT	sklearn.ensemble.GradientBoostingClassifier()
10	NB	sklearn.naive_bayes. GaussianNB()

The precision (Pre), accuracy(Acc), the area under the curve score (Auc), F1-score (F1), and Recall are the five commonly used indexes to evaluate classifiers (48). Generally, the higher the index value is, the more predictive the model is. Therefore, we applied these indexes to calculate each feature set's composite score (*CS*) to evaluate the ability of the feature set to classify HA from NA. We designed *CS* as the result of the coefficient times the mean score of the five indexes on the ten learning models. The coefficient was the average score of the five indexes on the models of all features obtained by methods in the same category [seen in Equation (2)].


(2) $$\begin{array}{l} CS\left(F_{method}\right)=H_{type}\frac{1}{KM}\underset{k,m}{\sum }index\left(k,\text{mod}el\left(m,F_{method}\right)\right) \end{array}$$

(3) $$\begin{array}{l} H_{type}=\frac{1}{KM}\underset{k,m}{\sum }index\left(k,\text{mod}el\left(m,F_{type}\right)\right) \end{array}$$

Wherein *K* and *M* are the total numbers of indexes and learning models, respectively, *K* = 5, *M* = 10, and k∈{Pre, Acc, Auc, F1, Recall}, m∈{SVM, DT, Ada, NN, RF, KNN, LR, DA, GBDT, NB}; *index (k, model(m, F*_*method*_*))* represents the *kth* index of the *mth* model fitted by *F*_*method*_; *H*_*type*_ is the coefficient of the *F*_*method*_, and *type* is the category to which the *method* belongs, *type*∈{FI, SIF, STF, SSL}.

We used the 13 *F*_*method*_ to perform tenfold cross-validation on the ten learning models for computing the Pre, Acc, Auc, F1, and Recall. During the tenfold cross-validation, the StratifiedKFold function imported from sklearn package was used to ensure the same proportion of NA and HA samples in the training and test sets. Besides, the *CS* measured according to Equations (2)–(3) were subsequently used to determine the top six feature sets F_top6_.

##### Verifying the ability to identify the degree of stroke in the brain tissue

Since the feature sets are obtained entirely on pure ischemic and normal tissue, the appearance of these features is worth studying when the tissue is impure, in the case of the region containing both normal and abnormal tissue. Therefore, this study further explored the relationship between the proportion of abnormal tissue in the brain and the representation of radiomics feature sets.

To expand the datasets, we split the brain into left and right sides and merged the data from both sides for an adequate analysis. Then, 160 samples can be generated from 80 images. First, for the process of verifying, we segmented the brain into left and right by split function in python 3.6. Secondly, the features in *F*_*top*6_of the middle S slices in the two sides of brain tissue were computed by Radiomics technology; S was 3, 4, and 5 in this study. Specifically, we extracted the *F*_*top*6_from the middle three, four, and five layers from DSC-PWI data. And then, the labels, representing whether the volume proportion of the ischemic region in these S slices of brain tissue was beyond the set reference threshold (*RT*), were made according to the results of the Rapid software. The label was 1 when the volume proportion of the ischemic region in the S slices was more than *RT* and 0 in the opposite situation. In this study, the *RT* was a sliding variable that came from the set starting at 0, ending at 0.39, and spaced at 0.01, *RT*∈{0, 001, 0.02, …, 039}. Therefore, *F*_*top*6_in each S were configured with 40 label groups, and each one in these 120 (40 ×3) combinations was regarded as *F*_*RT*_*S*_. Then, for each *F*_*RT*_*S*_, the best feature selection method concluded above was used to extract matched features with labels from the corresponding *F*_*top*6_, and the extracted results were defined as *F'*_*RT*_*S*_. Finally, tenfold cross-validation was performed on the ten models introduced in section 2.2.4 (A) with the *F'*_*RT*_*S*_. As *RT* gradually increases from 0 to 0.39, the proportion of ischemic area in the middle S slices will grow. Therefore, the test in this step could verify the ability to recognize the presence of stroke in differentiated degrees of ischemia. In this section, we also got the five indexes to evaluate the performance of *F'*_*RT*_*S*_ on each model.

## Results

Results are provided in three parts, including extracted significant radiomics features, selected outstanding features, and the performance of the selected feature sets. The details are shown in the following.

### Extracted significant radiomics features

**Figure 2:**
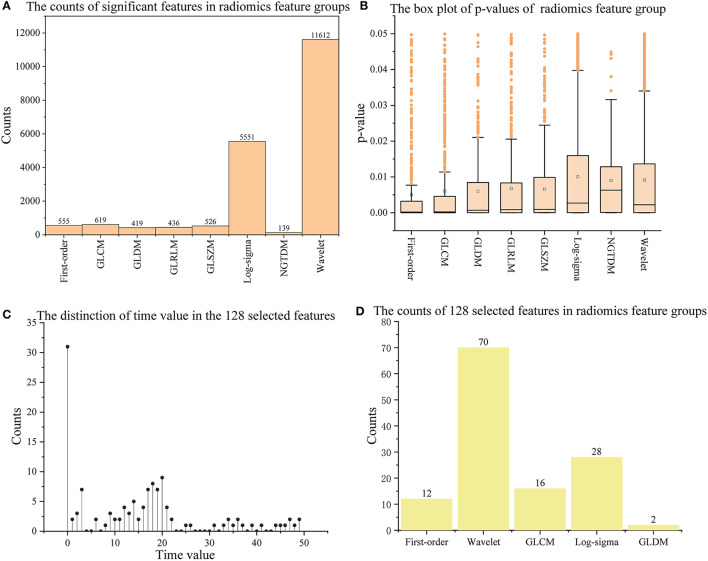
The information on significant features and 128 outstanding features. **(A,B)** Show the counts and *p-*values of significant features in each radiomics feature group; **(C,D)** show the time values of the 128 selected features and their counts in each radiomics feature group. The orange box in **(B)** indicates the distribution range of 25–75% *p-*values; The long horizontal line '—' above the box indicates 1.5 times the interquartile range value (1.5 IQR), and the discrete points above the short horizontal line are abnormal points.

Of all the 65,800 features computed by radiomics technology, in 19857 (30.2%) significant features were extracted with the *T-*test operation. Figures 2A,B show the *p-*value distribution of each radiomics feature group, and Table 4 illustrates their statistics. Features in the Shape of the nine radiomics groups were insignificant. However, the Wavelet and Log-sigma had the most salient features of 11,612 and 5,551, and their *p-*values ranged from 0.0092 ± 0.013 (mean ± std) and 0.01 ± 0.0138, respectively. The NGTDM group had minor significant features of 139, with *p-*values of 0.0090 ± 0.0107. The significant features in GLCM, Firs*t-*order, GLRLM, GLSZM, GLRLM, and GLDM were from 419 to 619, with *p-*values of nearly 0.006 ± 0.011. Combining Figures 2A,B, it can be seen that among all feature groups, the *p-*values of significant features in the eight radiomics feature groups can reach 0.05 at most. In addition, with the increasing number of significant features, the distribution range of them will decrease. That is, the distribution of the *p-*values excluding outliers will become more concentrated.

**Table 4:** The statistics of significant Radiomincs features groups.

**Feature Group**	**Number of features**	**Mean (*p-*value)**	**Std (*p-*value)**	**Minimum (*p-*value)**	**Median (*p-*value)**	**Maximum (*p-*value)**
Firs*t-*order	555	0.0050	0.0105	<0.0001	0.0002	0.0497
GLCM	619	0.0060	0.0114	<0.0001	0.0002	0.0499
GLDM	419	0.0060	0.0104	<0.0001	0.0007	0.0497
GLRLM	436	0.0068	0.0118	<0.0001	0.0009	0.0498
GLSZM	526	0.0066	0.0104	<0.0001	0.0009	0.0496
Log-sigma	5551	0.0100	0.0138	<0.0001	0.0027	0.0500
NGTDM	139	0.0090	0.0107	<0.0001	0.0063	0.0449
Wavelet	11612	0.0092	0.0130	<0.0001	0.0022	0.0500

### Selected outstanding features

With the 13 feature selection methods, 128 outstanding features were selected and renamed by combining the letter F and serial number (see Appendix A in Supplementary material). As an analysis result, the 128 features included 70 Wavelet features, 2 GLDM features, 16 GLCM features, 12 Firs*t-*order features, and 28 Log-sigma features (seen in Figures 2C,D). In addition, we computed four attributes between the label and each feature, including the coefficient of determination (R squared) based on the Pearson coefficient, *p-*value, Gain, and Gain ratio. The following were details described according to every single method.

In the methods based on FI (seen in Table 5), 64 excellent features with *p-*values = 0.009 ± 0.014, R squared = 0.090 ± 0.067, Gain = 0.077 ± 0.05 and Gain ratio=0.112 ± 0.072 were chosen, wherein CMIM, MM, JMI selected 20 features, respectively; MRMR and MIFS selected 18 features, respectively. Besides, the features in the five feature sets were highly repeatable.

**Table 5:** The counts of features and four attributes of 13 *F*_*method*_.

**Type**	**Method**	**Counts of Features**	**R squared**	**p_value**	**Gain**	**Gain ratio**
FI	CMIM	20	0.11 ± 0.657	0.004 ± 0.011	0.089 ± 0.048	0.129 ± 0.069
	MIM	20	0.116 ± 0.074	0.008 ± 0.015	0.102 ± 0.065	0.147 ± 0.093
	JMI	20	0.077 ± 0.073	0.014 ± 0.016	0.058 ± 0.036	0.083 ± 0.051
	MRMR	18	0.064 ± 0.039	0.009 ± 0.01	0.062 ± 0.032	0.089 ± 0.047
	MIFS	18	0.078 ± 0.044	0.006 ± 0.009	0.066 ± 0.032	0.095 ± 0.046
SIF	Fisher	4	0.603 ± 0.004	<0.0001	0.474 ± 0.009	0.684 ± 0.013
	ReliefF	12	0.538 ± 0.065	<0.0001	0.389 ± 0.078	0.561 ± 0.112
	LS	6	0.124 ± 0.190	0.013 ± 0.018	0.102 ± 0.142	0.147 ± 0.204
STF	FS	7	0.592 ± 0.015	<0.0001	0.464 ± 0.022	0.670 ± 0.032
	TS	11	0.582 ± 0.018	<0.0001	0.430 ± 0.064	0.621 ± 0.093
SSL	Alpha	11	0.199 ± 0.167	0.006 ± 0.014	0.145 ± 0.115	0.209 ± 0.166
	Lasso	16	0.280 ± 0.146	<0.0001	0.197 ± 0.106	0.284 ± 0.152
	MCFS	20	0.087 ± 0.062	0.011 ± 0.012	0.069 ± 0.036	0.1 ± 0.052

The methods in SIF selected 18 features (seen in Table 5). The attributes of them were R squared = 0.4 ± 0.232, *p-*values = 0.004 ± 0.012, Gain =0.293 ± 0.171, and Gain ratio=0.423 ± 0.247, respectively. Of these 18 features, only four came from the *F*_*Fisher*_, while *F*_*Lap*_ and *F*_*ReliefF*_ contributed 6 and 16 features. The features in these sets had the lower *p-*values and the higher R squared, Gain, and Gain ratios.

In the methods based on STF (seen in Table 5), 11 features were obtained. These 11 features all belong to the *F*_*TS*_, and *F*_*FS*_ included only 6 of them. In addition, the R squared, Gain and Gain ratio ranged from 0.582 ± 0.018, 0.43 ± 0.064, and 0.621 ± 0.093, respectively. And the *p-*values of them were <0.0001. Besides, *F*_*FS*_ and *F*_*TS*_ got similar results on the four attributes, among which the index value of *F*_*FS*_ was slightly higher than that of *F*_*TS*_.

In the methods based on SSL (seen in Table 5), there were 47 selected features. The features in the three feature sets were scattered and independent. *F*_*MCFS*_ screened out 20 features independent of *F*_*Lasso*_ and *F*_*Alpha*_, while *F*_*Lasso*_ and *F*_*Alpha*_ shared a few members in common. The 47 features configured with R squared = 0.179 ± 0.148, *p-*values = 0.006 ± 0.011, Gain = 0.13 ± 0.101, and Gain ratio=0.188 ± 0.146.

### Performance of feature sets

In this study, we evaluated the 13 feature sets in two aspects. One was to identify HA and NA, and the other was to determine the proportion of ischemic lesions in brain tissue.

#### The performance of identifying HA and NA

**Figure 3:**
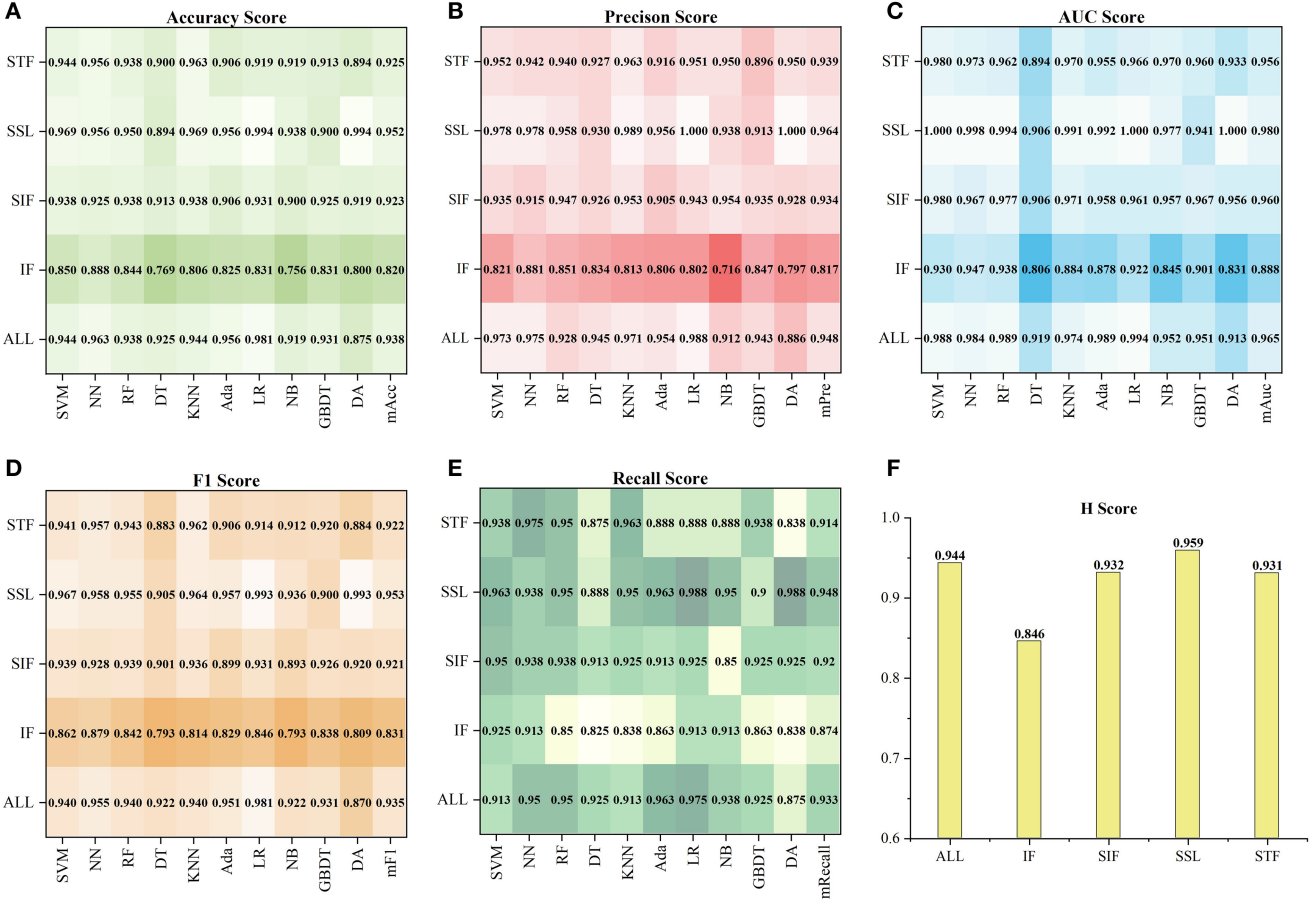
The performance of each *F*_*type*_and *F*_*all*_on the ten models. **(A–E)** Show the five indexes (Acc, Pre, Auc, F1, Recall) of *F*_*type*_and *F*_*all*_, and **(F)** show the coefficients *H*_*type*_of them.

Based on the tenfold cross-validation results on the ten models, we calculated the five indexes of the five *F*_*type*_ and 128 selected features *F*_*all*_ on the ten models and then got their *H*_*type*_. Figure 3 shows their performance in detail. According to the mean of five indexes (mAcc, mAuc, mPre, mF1, mRecall), SSL got the best score of mAcc = 0.952, mPre = 0.964, mAuc = 0.980, mF1 = 0.953 and mRecall= 0.948, while FI got the lowest score of mAcc = 0.82, mPre = 0.817, mAuc = 0.888, mF1 = 0.831 and mRecall = 0.874. Besides, SIF and STF got similar scores, and SIF was slightly better than STF. The results also showed that the performances of *F*_*all*_ were lower than that of *F*_*SSL*_, but generally better than other feature sets, which means that although the total features achieved good performance, it was still slightly inferior to the combination of the best feature sets. In addition, the coefficients *H*_*type*_ of *F*_*type*_ were computed according to Equation (3). As a result, *F*_*SSL*_ obtained the highest coefficient of 0.959, and the coefficient values of *F*_*all*_, *F*_*SIF*_, *F*_*STF*_, and *F*_*FI*_ decreased successively, which were 0.944, 0.932, 0.931, and 0.846.

**Figure 4:**
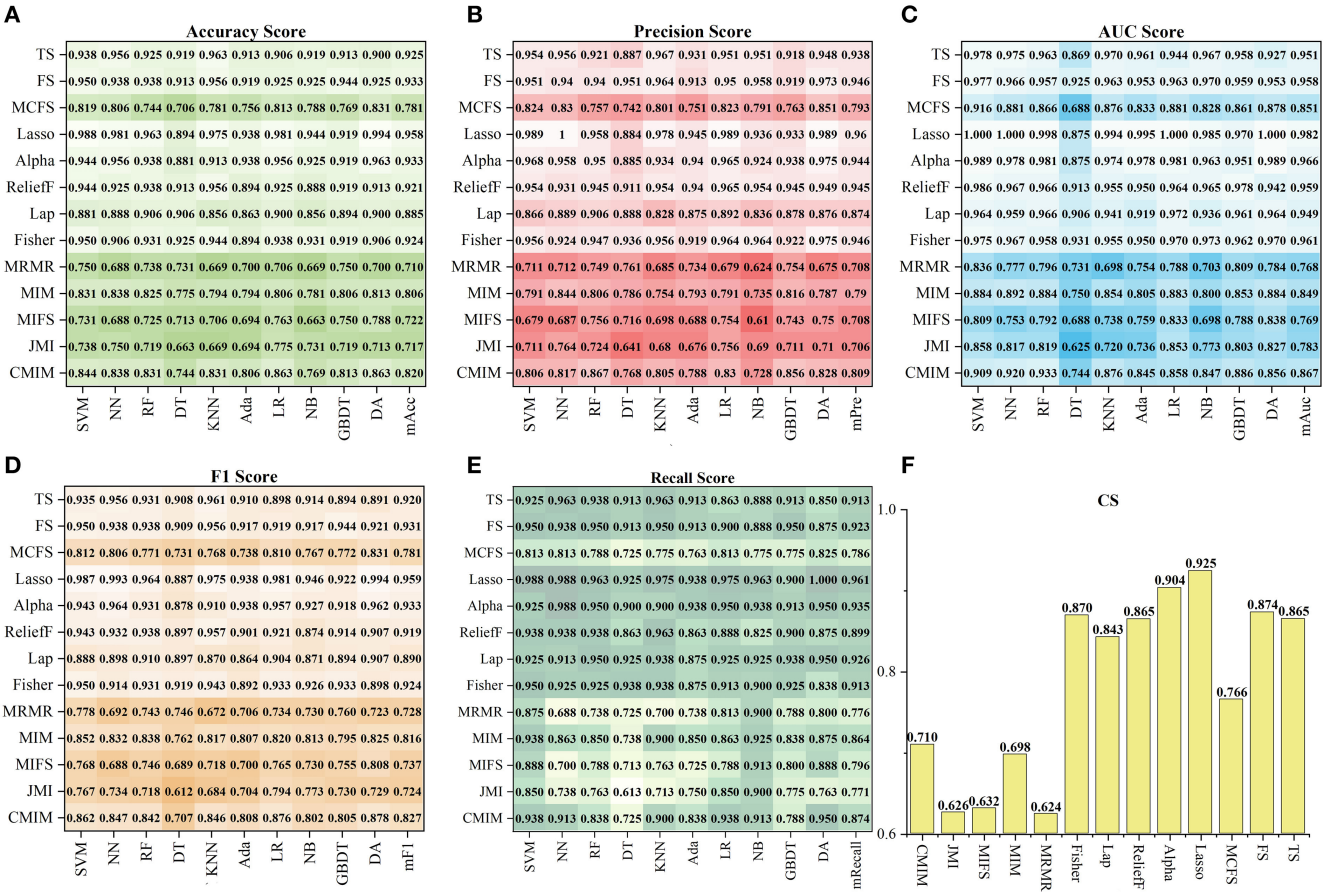
The performance of 13 feature sets on the ten models. **(A–E)** Show the five index (Acc, Pre, Auc, F1, Recall) results, and **(F)** shows the corresponding CS.

Figure 4 shows the tenfold cross-validation scores of 13 feature sets on the ten models. For the 13 feature sets, the mAcc, mPre, mAuc, mF1, and mRecall were 0.849, 0.851, 0.893, 00853, and 0.872, respectively. And the *CS* of them were from 0.624 to 0.925. In general, the performance of a single feature set was consistent with the result of the *F*_*type*_to which it belongs. Similar to statistics by types of features, the feature sets in *F*_*SSL*_ performed better than those in the other *F*_*type*_, and sets in *F*_*FI*_ got a result that left much for improvement. Specifically, using *CS* as a reference (seen in Figure 4F), the best one was *F*_*Lasso*_(*CS* = 0.925) *in the F*_*SSL*_, and *F*_*Alpha*_ got a comparable *CS* of 0.904. In particular, *F*_*Lasso*_ achieved an Auc of 1 on multiple models. In contrast, *F*_*MRMR*_, *F*_*MRMR*_, and *F*_*MIFS*_ in *F*_*FI*_ performed relatively poorly. The other feature sets scored differently, ranging from 0.70 to 0.874. In general, the top six feature sets *F*_*top*6_with the highest *CS* were *F*_*Lasso*_, *F*_*Alpha*_, *F*_*FS*_, *F*_*Fisher*_, *F*_*TS*_, and *F*_*ReliefF*_, including 41 features. Besides, the Lasso algorithm became the best method for subsequent feature selection processing based on the highest *CS*.

#### The ability to identify the proportion of ischemic stroke

**Figure 5:**
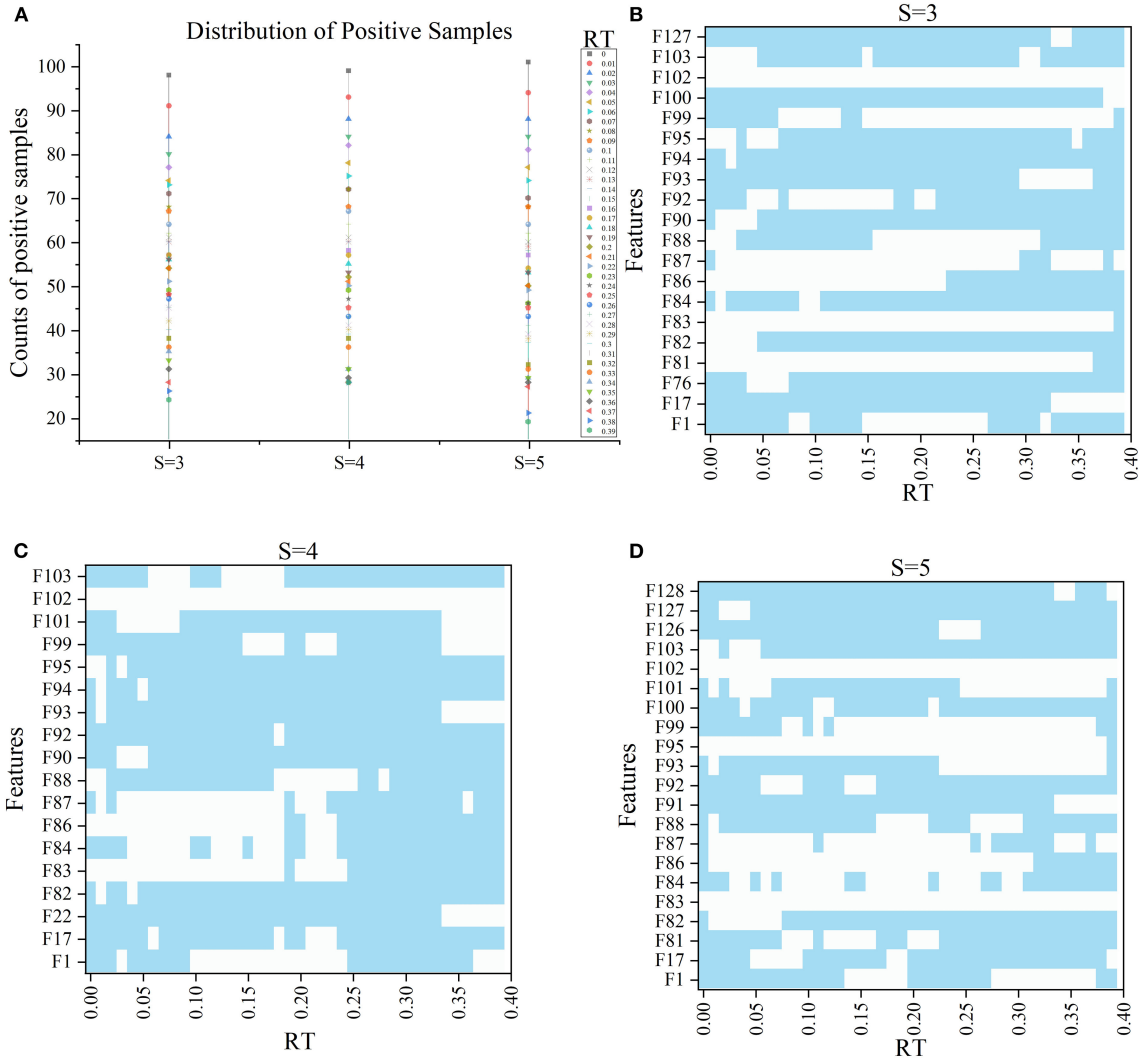
The information of samples and *F*${}_{RT\text{\_}S}^{{}^{\prime }}$. **(A)** Shows the distribution of positive samples with ranging *S* and *RT* and the features in *F*${}_{RT\text{\_}S}^{{}^{\prime }}$, and **(B–D)** show the selected features under different RT values when *S* = 3, 4, and 5 respectively, wherein blue indicates that the corresponding features are selected.

In the 120 *F*_*RT*_*S*_ formed by the *F*_*top*6_ of the three *S* slices under 40 *RT*, positive samples (label=1) indicated that ischemic stroke volume greater than *RT* differed. Figure 5A shows the distribution of positive samples in each *S* with different *RT* values. In each case, positive samples decreased gradually as *RT* values increased. In general, the ratio of positive samples in 160 patients ranged from 15 to 61.25% in *S* = 3, 17.5–61.88% in *S* = 4, and 11.88–63.13% in *S* = 5. When *RT* was in the range of 0–0.04, the proportion between positive and negative samples was >1:1; when *RT* was in 0.05–0.1, the proportion was about 2:3; when *RT* was in 0.11–0.25, the proportion was nearly 1:3, and when *RT* was >0.25, the proportion was <0.3.

**Figure 6:**
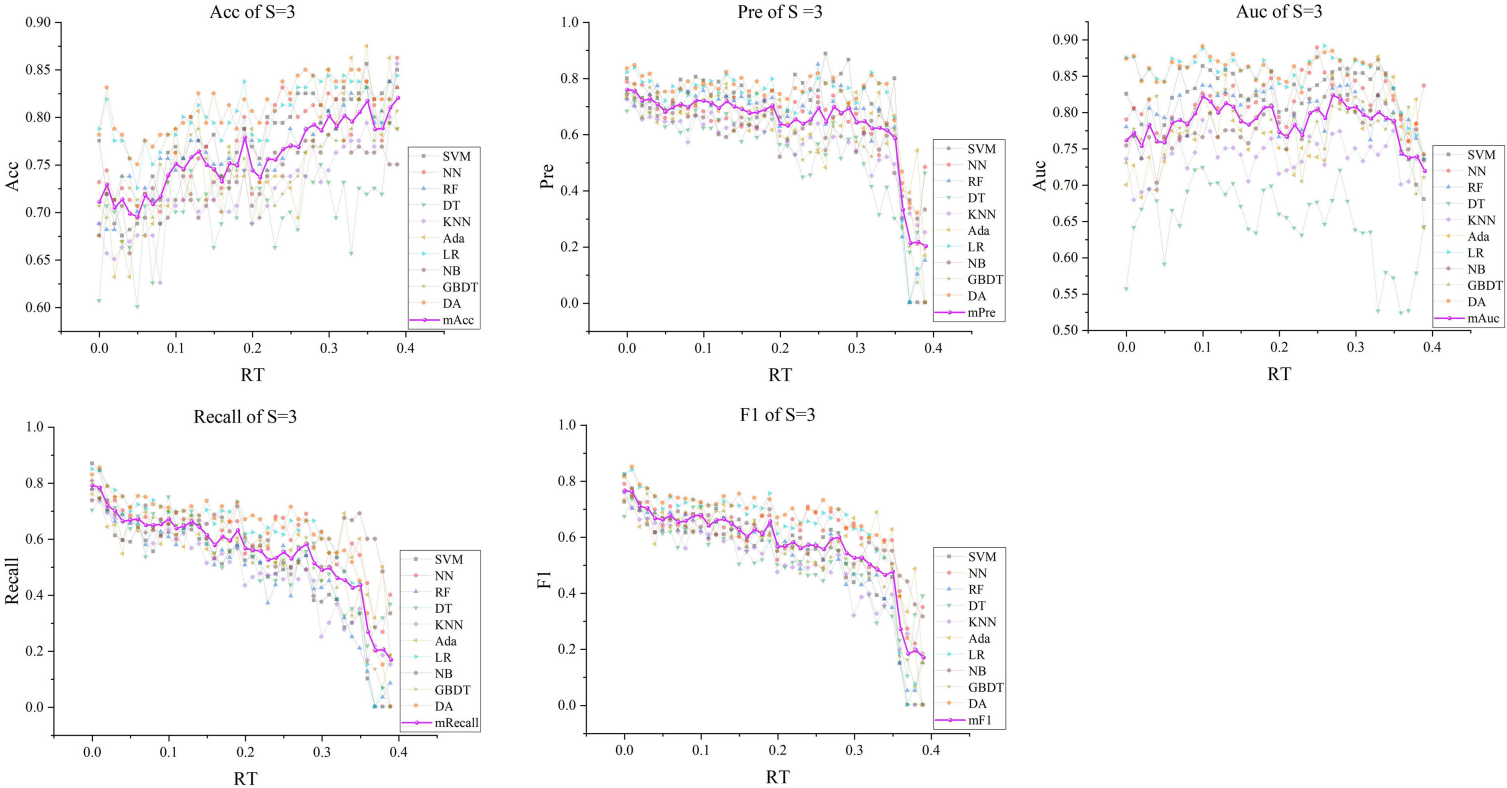
The five indexes of *F*${}_{RT\text{\_}S}^{{}^{\prime }}$ with *S* = 3 on the ten models, wherein the dark purple lines represent the mean indexes (mAcc, mPre, mAuc, mF1, mRecall), and the other colors represent the performance of the ten models.

**Figure 7:**
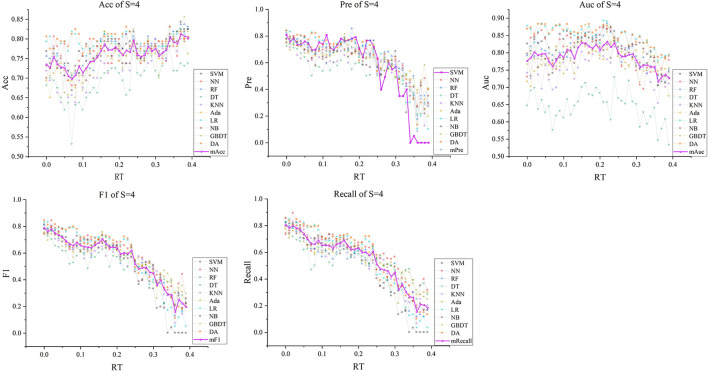
The five indexes of *F*${}_{RT\text{\_}S}^{{}^{\prime }}$ with *S* = 4 on the ten models, wherein the dark purple lines represent the mean indexes (mAcc, mPre, mAuc, mF1, mRecall), and the other colors represent the performance of the ten models.

**Figure 8:**
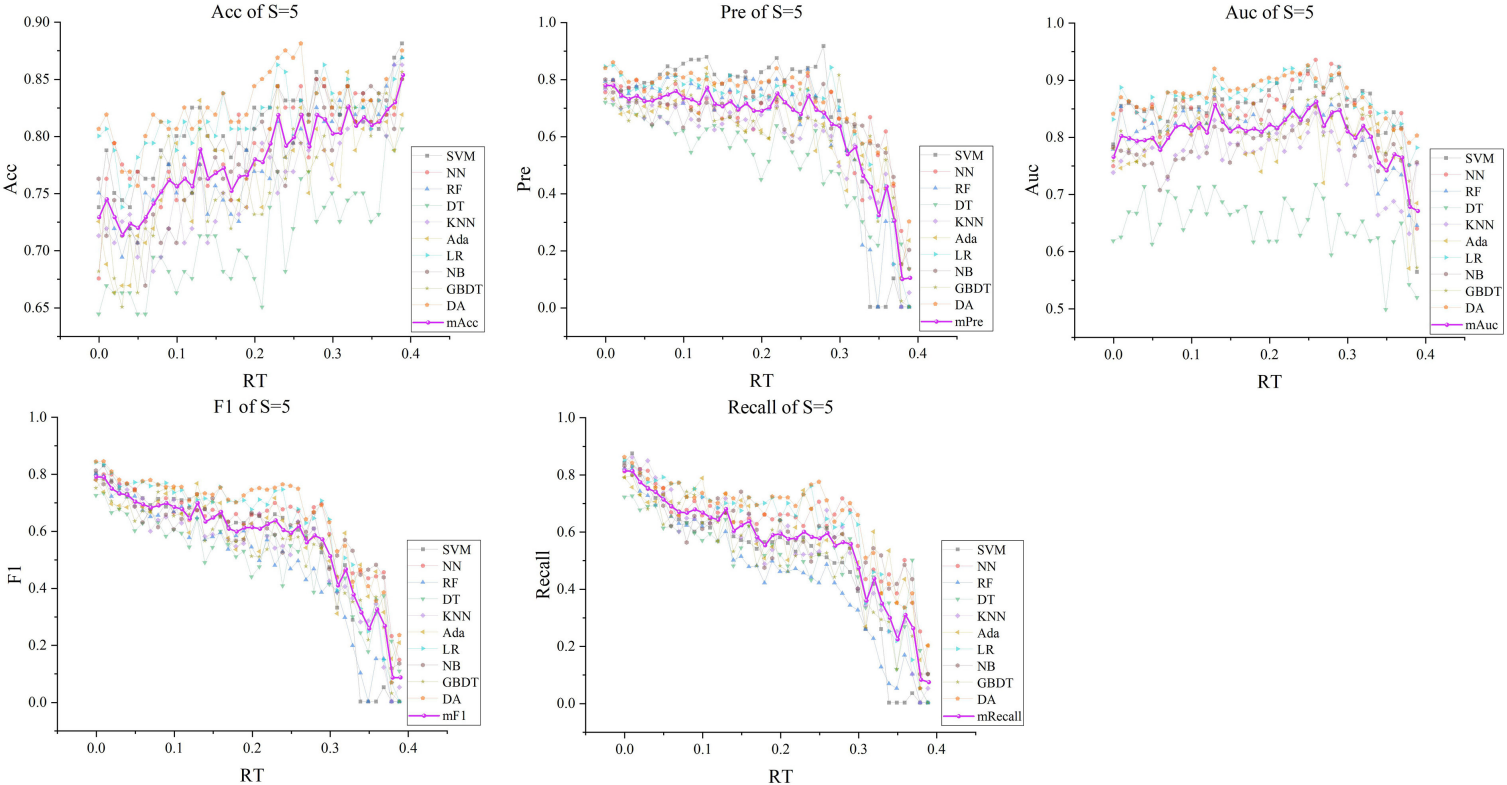
The five indexes of *F*${}_{RT\text{\_}S}^{{}^{\prime }}$ with *S* = 5 on the ten models, wherein the dark purple lines represent the mean indexes (mAcc, mPre, mAuc, mF1, mRecall), and the other colors represent the performance of the ten models.

**Figure 9:**
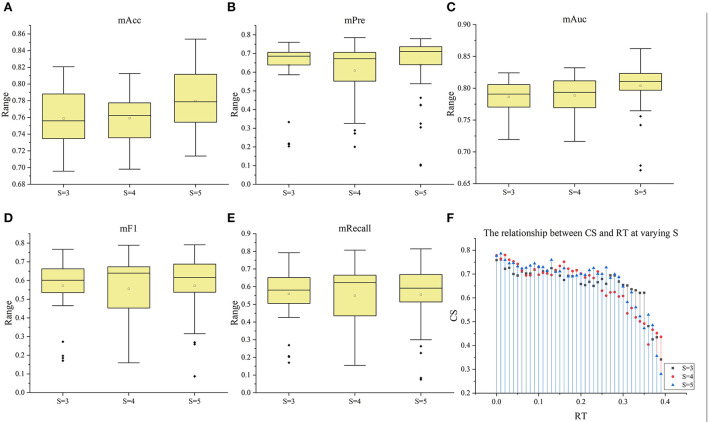
The box plots of the five index under the three situation (S = 3–5). **(A–E)** The box plots of mACC, mPre, mAuc, mF1, and mRecall in the three situations, respectively. **(F)** The relationship between CS and RT at varying S.

Subsequently, with *RT* from 0 to 0.39 and *S* from 3 to 5, we selected outstanding features *F*${}_{RT\text{\_}S}^{{}^{\prime }}$ by the Lasso algorithm. As a result, there were slight differences between the features in 140 *F*${}_{RT\text{\_}S}^{{}^{\prime }}$. There were 20 features in *F*${}_{RT\text{\_}3}^{{}^{\prime }}$, 18 in *F'*_*RT*_4_, and 21 in *F'*_*RT*_5_, and most of these features came from *F*_*Lasso*_ and *F*_*Alpha*_ (seen in Appendix A in Supplementary material). Figures 5B–D show the detailed features. We got the five indexes on the ten models by performing the tenfold cross-validation with the selected *F*${}_{RT\text{\_}S}^{{}^{\prime }}$. As Figures 6–8 show, whatever the S value was, with the increase of *RT*, the Acc of the ten models showed a gradual growth trend; Pre and Auc represented a state of steady first and then slow decline nearly at *RT*∈*[0.24, 0.3]*, while F1 and Recall gradually decreased. Among them, the mAcc ranged from 0.6 to 0.875 in *F*${}_{RT\text{\_}3}^{{}^{\prime }}$, 0.531 to 0.856 in *F*${}_{RT\text{\_}4}^{{}^{\prime }}$, 0.644 to 0.881 in *F*${}_{RT\text{\_}5}^{{}^{\prime }}$; the mAuc ranged from 0.523 to 0.892, 0.533 to 0.893, 0.497 to 0.935; and the mPre ranged 0 to 0.888, from 0 to 0.856, 0 to 0.917; the mF1 ranged from 0 to 0.85, 0 to 0.845, 0 to 0.844, and mRecall from 0 to 0.87, from 0 to 0.896, 0 to 0.874, respectively. Furthermore, the *CS* of *F*${}_{RT\text{\_}S}^{{}^{\prime }}$ stayed stable and then dropped rapidly. And the *CS* ranged from 0.759 to 0.341 in *F*${}_{RT\text{\_}3}^{{}^{\prime }}$, from 0.78 to 0.437 in *F*${}_{RT\text{\_}4}^{{}^{\prime }}$, and from 0.786 to 0.28 in *F*${}_{RT\text{\_}5}^{{}^{\prime }}$. According to Figure 9, the drop point was at the stage when *RT* was >0.25, and that of *S* = 3 was later than that of *S* = 4 and 5.

## Discussion

**Figure 10:**
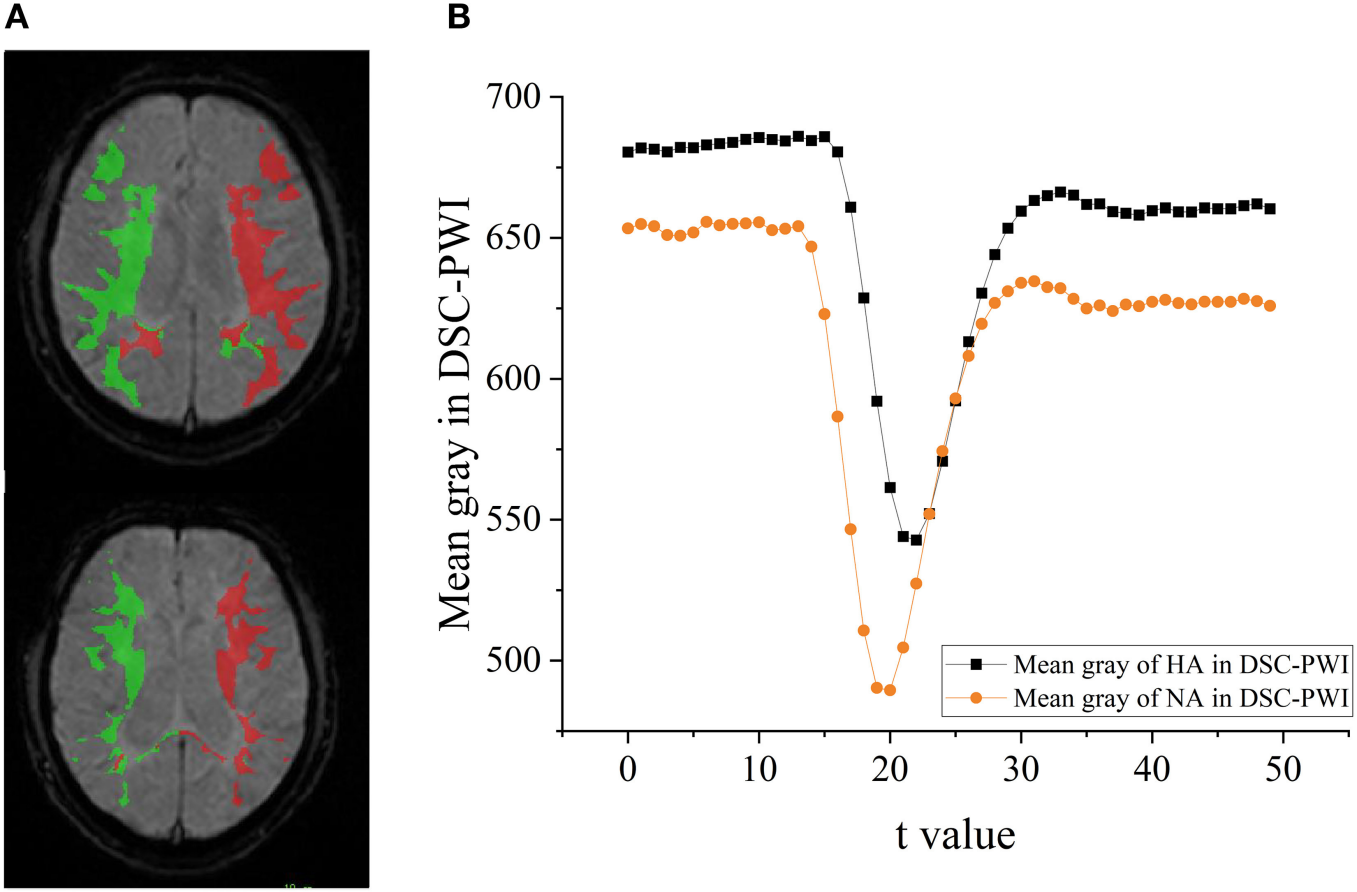
The difference of HA and NA in the DSC-PWI image. **(A)** The ROIs of HA and NA in the DSC-PWI image, the HA is shown in red and the NA is shown in green. **(B)** The mean time-intensity curve *I*(*t*) of HA and NA, the black represents the mean *I*(*t*) of HA, and the orange is that of NA.

An ischemic stroke is a vascular event characterized by reducing regional blood flow. Few studies explored the changes among DSC-PWI images in the time dimension, although the parameter *T*_*max*_ obtained from them was commonly used to discriminate HA and NA. Some studies (49–51) have shown that the time-intensity curve of HA in the DSC-PWI images of patients with ischemic stroke has a much smaller brightness decrease than the curve of NA (seen in Figure 10). Therefore, the data of DSC-PWI in the time dimension are correlated with the blood flow state of brain tissues to a certain extent. This study successfully extracted multi-level feature selection processing and the radiomics features distinguishing HA and NA from DSC-PWI. Of all the methods, the *F*_*Lasso*_ reached the best *CS* of 0.925, and the five indexes were mAcc of 0.958, mPre of 0.96, mAuc of 0.982, mF1 of 0.959, and mRecall of 0.96. Besides, we effectively verified the ability of these features to evaluate the ischemic area ratio in the brain. According to the results, with the increase of the proportion of ischemic tissue, the mAcc increased, while Pre stabilized and then decreased. And the best Pre and Acc can reach 0.888 and 0.863. In general, the radiomics features of 3D images in the time dimension of DSC-PWI have an optimistic ability to distinguish normal brain tissue from abnormal brain tissue and indicate the proportion of ischemic tissue in brain tissue.

This study used 13 feature selection methods with different preferences to obtain outstanding features. As a result, there were 128 excellent features selected from the original 65,800 radiomics features. Their time values are mainly concentrated at the initial moment (0–3), the stage through which the contrast agent passes (17–22), and a few features located at the end of the reaction (time >30) (seen in Figure 2C). The results indicate that the initial intensity of the tissue, as well as the amount of intensity change, and the time producing the change, are essential to distinguish between normal and abnormal tissues. Besides, the features of Shape, GLRLM, GLSZM, and NGTDM were missing in the 128 selected features (seen in Figure 2D). This means that the shape, gray of neighboring voxels, and length in the number of pixels with the same gray make little contribution to characterizing the changing of blood flow, and features in the other groups are significant. Among 13 feature sets, *F*_*Lasso*_ and *F*_*Alpha*_ in *F*_*SSL*_ achieved the best *CS* of 0.925 and 0.904, while *F*_*FI*_ performed worst, and *F*_*SIF*_ and *F*_*STF*_performed in the middle (seen in Figure 4). From the four attributes (p-value, R squared, Gain, and Gain ratio), the *p-*value of *F*_*FI*_ is more significant than the others. In contrast, the R squared, Gain, and the Gain ratio are less than the others, suggesting that the effect of features extracted by *FI* may not be ideal. In addition, the feature selection methods (CMIMI, JMI, MIFS, MIM, and MRMR) in the *FI* mainly select features based on the conditions of information entropy, redundancy, and similarity between radiomics features to obtain feature sets. Among the 65,800 original radiomics features, there may be a large number of features that meet the above screening conditions. However, in this study, the number of selected features of the feature selection method is limited to 10. Although this study ranks features according to their scores, the selected features may not be complete, resulting in the unsatisfactory performance of F_*FI*_. This can be further improved and verified in subsequent experiments. Different from *FI*, other feature selection methods in *SIF, STF*, and *SSL* selected features through the linear relationship, contributions, and statistical scores between features and sample categories. When sorting features with scores, excellent features will be selected first, so it is reasonable that they got a relatively higher performance than *FI*. In detail, For the feature sets in *F*_*SIF*_, the attributes of *F*_*LS*_ have less correlation and information than the others, and they got a matching result that the CS of *F*_*Fisher*_ and *F*_*ReliefF*_ are better than *F*_*Lap*_. *F*_*FS*_ and *F*_*TS*_ in *F*_*STF*_ got similar attributes and achieved the closer CS of 0.874 and 0.865. For feature sets in *F*_*SSL*_, although *F*_*Lasso*_ and *F*_*Alpha*_ obtained a minor R squared, Gain and Gain ratio than feature sets in *F*_*STF*_and *F*_*SIF*_, they achieved the best performance. For the long term, Lasso has been used to select excellent features and has been validated in the fields of classification (52–54), prediction (55, 56), and survival analysis (57, 58). In this study, Lasso got the best feature set *F*_*Lasso*_ to prove its competence in screening features. Thus, although the lower *p-*value, higher correlation, information gain, and information gain ratio can achieve a better classification result to a certain extent, they cannot be used as complex indicators to evaluate their effectiveness. The 13 *F*_*method*_ are a great deal of diversity, and these selected features are highly significant. No matter what selection method is used, they can obtain the characteristics of DSC-PWI from the aspects of intensity variation, drop time of intensity, initial state, and recovery state.

Furthermore, this study analyzed the classification ability of radiomics features in different proportions of ischemic lesions. With the increase of *RT*, the region of ischemic tissue increases, and the difference between features whose *RT* is above the set threshold and those of the opposite class decreases. When *RT* was <0.25, regardless of *S* = 3, 4, or 5, the Acc and Pre can reach >0.8. However, when *RT* was >0.25, the performance will decrease with the increase of *RT*. On the one hand, the decline of these two indexes may be due to the imbalance in the proportion of positive and negative samples when *RT* reaches 0.25. If sufficient data are available, in-depth reason analysis can be performed in the future. Nevertheless, these results demonstrate that the radiomics features can effectively distinguish normal tissue from ischemic tissue, provide support for the differentiation of volume proportion of ischemic lesions and provide information for clinical guidance.

In addition, ten models with different principles were used to verify the performance of selected features. The ten models included regression models (LR, NB), nonlinear classifiers (SVM, DT, RF), linear classifiers (KNN, DA), ensemble models (Ada, GBDT), and neural networks (NN). According to the classification results of these models, the classification effect of selected features can be verified comprehensively. Figures 4, 6–8 show little difference in the performance of the same feature set in different models. Still, there is a significant difference in the performance of different feature sets in the same model, and SVM, LR, NN, RF, and DA performed better than the others. For the classification of HA and NA, SVM performed best in almost all feature sets, with an mAUC of 0.929. In particular, the Auc, Pre, Acc, F1, and Recall of CSVM_Lasso were all >0.987. Using a nonlinear kernel 'RBF' in SVM, the nonlinear relationship between the selected radiomics features and the target (stroke tissue or not) can be found, thus obtaining accurate classification results. Besides, DA, RF, NB, LR, and NN also achieved satisfactory results. Regarding identifying the proportion of ischemic stroke, DA and SVM also performed better than the other models in all three situations, RT_2, RT_3, and RT_3. Although the performance of different models on the same feature set and the same situation had good consistency, SVM was a better choice in both evaluation tasks, classifying HA and NA and identifying the proportion of ischemic stroke. Besides, we used *CS* computed by the mean indexes (mAcc, mPre, mAuc, mF1, and mRecall) of the ten models as the benchmark for the evaluation to reasonably analyze their performance. Depending on the diversity features, the 13 *F*_*method*_ acquired different *CS* ranging from 0.624 to 0.925, F${}_{RT\text{\_}3}^{{}^{\prime }}$, F${}_{RT\text{\_}4}^{{}^{\prime }}$, and F${}_{RT\text{\_}5}^{{}^{\prime }}$ got *CS* in [0.34,0.76], [0.40, 0.78] and [0.28,0.78], respectively. On the one hand, the strong robustness and applicability of the Lasso algorithm can be proved by the fact that, although the features extracted by the algorithm were slightly different under different *RT* values, the extracted features generally achieved stable performance. On the other hand, the selected radiomics features at different slices have little influence on the classification results, but the proportion of ischemic tissue does.

There are some limitations to this study. First, the size of the datasets is relatively small, and all data come from a single hospital, which may lead to biased results and a lack of generalizability. To address the limitation, we segmented hypoperfusion areas (HA) from DSC-PWI images and defined normal tissue in the symmetrical areas of HA as NA in making ROIs. This way, one group of HA and NA can be generated from one DSC-PWI image. This way, the double samples (160) can be obtained from 80 DSC-PWI images, and the positive and negative sample sizes are equal. The expanded balanced samples can help extract accurate features, and the sample imbalance can be reduced when classifying NA and HA. Besides, when evaluating the performance of the selected features in section Performance evaluation of the selected features, the tenfold cross-validation was performed to reduce the influence of sample size. The composite scores (CS) were computed to obtain reliable results. Second, the feature selection methods, optimal features, and learning models can be further optimized. This paper uses various existing learning models to verify the classification performance. Although the results have shown some features such as *F*_*Lasso*_ and *F*_*Alpha*_ had achieved excellent performance, the further optimization of the models, such as deep learning and transferred learning, can be regarded as one of the future works. The ischemia area ratio classification needs to be further improved. The results in this study do not mean that the models can be used alone for stroke treatment decision-making. Instead, it should be considered a support tool for stroke treatment guidance. We will validate our improved method's performance with more data before applying it to clinical trials in future work.

## Conclusions

This study used prominent radiomics features extracted from 3D images in the DSC-PWI time series to explore their ability to classify HA and NA and recognize the proportion of ischemic lesions in brain tissue. The 13 *F*_*method*_ achieved the *CS* ranging from 0.624 to 0.925 in distinguishing HA from NA. The *F*_*Lasso*_ in the 13 *F*_*method*_ performed best with mAcc of 0.958, mPre of 0.96, mAuc of 0.982, mF1 of 0.959, and mRecall of 0.96. Besides, the 120 *F*${}_{RT\text{\_}S}^{{}^{\prime }}$ reached the best *CS* of 0.786 in identifying the proportion of the ischemic region, and the best Acc and Pre reached 0.888 and 0.863, respectively. In general, the combination of various radiomics features accurately reflected the varying degrees of changes in cerebral blood flow in the initial state, the contrast agent response stage, and the recovery stage. For classifying the proportion of ischemic areas, the classification effect is relatively stable when RT is <0.25. Otherwise, when *RT* was >0.25, the accuracy will gradually decrease as its increases. Further future research should be conducted on excellent feature extraction, feature combination, model optimization, and comprehensive verification.

## Data availability statement

The original contributions presented in the study are included in the article/Supplementary material, further inquiries can be directed to the corresponding author/s.

## Ethics statement

The studies involving human participants were reviewed and approved by Shanghai Fourth People's Hospital Affiliated to Tongji University School of Medicine, Shanghai 200434, China. The patients/participants provided their written informed consent to participate in this study. Written informed consent was obtained from the individual(s) for the publication of any potentially identifiable images or data included in this article.

## Author contributions

YLu, WL, and YK conceived of the idea and encouraged YG to investigate and supervised the findings of this work. YLi provided the datasets. YG, YY, FC, MF, XZ, WQ, and LC developed the theory and performed the computations. YG, QL, XM, and YLi compared the analytical methods. YG, LL, and XZ analyzed and discussed results. YG, YLi, and YY wrote and corrected the manuscript with input from all authors. All authors discussed the results and contributed to the final manuscript.

## Funding

The study was supported by the Stable Support Plan for Colleges and Universities in Shenzhen, China (SZWD2021010), the Scientific Research Fund of Liaoning Province, China (JL201919), and the National Natural Science Foundation of China (62071311).

## Conflict of interest

Author LC was employed by Shenzhen Happy-Growing Intelligent CO., Ltd.

The remaining authors declare that the research was conducted in the absence of any commercial or financial relationships that could be construed as a potential conflict of interest.

## Publisher's note

All claims expressed in this article are solely those of the authors and do not necessarily represent those of their affiliated organizations, or those of the publisher, the editors and the reviewers. Any product that may be evaluated in this article, or claim that may be made by its manufacturer, is not guaranteed or endorsed by the publisher.
