# Supplementary material for: Radiomics features of DSC-PWI in time dimension may provide a new chance to identify ischemic stroke
Source: Front Neurol. 2022 Nov 4;13:889090. doi: 10.3389/fneur.2022.889090 (PMC9672479; doi:10.3389/fneur.2022.889090)
Supplement: Supplementary file 1 [file Table_1.DOCX]

Supplementary Material

**Appendix. A.** The information of all 128 selected features. The symbol “√” indicates that the feature is selected.

| Definition | Group | Radiomics Features | Method | *F_top6_* | *F^’^_RT_S_* | | |
| --- | --- | --- | --- | --- | --- | --- | --- |
|  |  |  |  |  | S=3 | S=4 | S=5 |
| F1 | First-order | original_firstorder_10Percentile_0 | CMIM; DISR; JMI; MIFS; MIM; MRMR; Alpha; | **√** | √ | √ | √ |
| F2 | Wavelet | wavelet-HHL_glszm_GrayLevelNonUniformityNormalized_0 | CMIM; JMI; MIFS; |  |  |  |  |
| F3 | Wavelet | wavelet-HHL_glszm_LowGrayLevelZoneEmphasis_0 | CMIM; |  |  |  |  |
| F4 | Wavelet | wavelet-HHL_glszm_GrayLevelVariance_1 | CMIM; |  |  |  |  |
| F5 | Wavelet | wavelet-HHL_glszm_HighGrayLevelZoneEmphasis_11 | CMIM; |  |  |  |  |
| F6 | Wavelet | wavelet-HHL_glszm_GrayLevelNonUniformityNormalized_12 | CMIM; |  |  |  |  |
| F7 | Wavelet | wavelet-HHL_glszm_GrayLevelVariance_17 | CMIM; |  |  |  |  |
| F8 | First-order | original_firstorder_90Percentile_0 | CMIM; DISR; MIM; |  |  |  |  |
| F9 | First-order | original_firstorder_Kurtosis_0 | CMIM; DISR; MIM; Alpha; | √ |  |  |  |
| F10 | First-order | original_firstorder_MeanAbsoluteDeviation_0 | CMIM; DISR; MIM; |  |  |  |  |
| F11 | First-order | original_firstorder_Mean_0 | CMIM; DISR; MIM; |  |  |  |  |
| F12 | First-order | original_firstorder_Median_0 | CMIM; DISR; MIM; |  |  |  |  |
| F13 | First-order | original_firstorder_RootMeanSquared_0 | CMIM; DISR; MIM; |  |  |  |  |
| F14 | First-order | original_firstorder_Skewness_0 | CMIM; DISR; MIM; |  |  |  |  |
| F15 | First-order | original_firstorder_Variance_0 | CMIM; DISR; MIM; |  |  |  |  |
| F16 | GLCM | original_glcm_Autocorrelation_0 | CMIM; DISR; MIM; |  |  |  |  |
| F17 | GLCM | original_glcm_ClusterProminence_0 | CMIM; DISR; MIM; Alpha; | √ | √ | √ | √ |
| F18 | GLCM | original_glcm_ClusterShade_0 | CMIM; DISR; MIM; |  |  |  |  |
| F19 | GLCM | original_glcm_ClusterTendency_0 | CMIM; DISR; MIM; |  |  |  |  |
| F20 | GLCM | original_glcm_Correlation_0 | CMIM; DISR; MIM; |  |  |  |  |
| F21 | GLCM | original_firstorder_Minimum_0 | DISR; MIM; |  |  |  |  |
| F22 | GLCM | original_firstorder_Range_0 | DISR; MIM; |  |  |  |  |
| F23 | GLCM | original_glcm_DifferenceEntropy_0 | DISR; MIM; |  |  |  |  |
| F24 | GLCM | original_glcm_DifferenceVariance_0 | DISR; MIM; |  |  |  |  |
| F25 | GLCM | original_glcm_JointAverage_0 | DISR; MIM; |  |  |  |  |
| F26 | GLCM | original_glcm_MCC_0 | DISR; MIM; |  |  |  |  |
| F27 | Wavelet | wavelet-HHH_glszm_GrayLevelNonUniformityNormalized_0 | JMI; MIFS; MRMR; |  |  |  |  |
| F28 | Wavelet | wavelet-HHH_glszm_GrayLevelNonUniformityNormalized_20 | JMI; |  |  |  |  |
| F29 | Wavelet | wavelet-HHH_glszm_GrayLevelNonUniformityNormalized_11 | JMI; |  |  |  |  |
| F30 | Wavelet | wavelet-HHL_glszm_GrayLevelNonUniformityNormalized_18 | JMI; |  |  |  |  |
| F31 | Wavelet | wavelet-HHL_glszm_HighGrayLevelZoneEmphasis_1 | JMI; |  |  |  |  |
| F32 | Wavelet | wavelet-HHL_glszm_HighGrayLevelZoneEmphasis_13 | JMI; |  |  |  |  |
| F33 | Wavelet | wavelet-HHL_glszm_HighGrayLevelZoneEmphasis_3 | JMI; MRMR; |  |  |  |  |
| F34 | Wavelet | wavelet-HHH_glszm_GrayLevelNonUniformityNormalized_16 | JMI; |  |  |  |  |
| F35 | Wavelet | wavelet-HHH_glszm_HighGrayLevelZoneEmphasis_18 | JMI; |  |  |  |  |
| F36 | Wavelet | wavelet-HHL_glszm_GrayLevelNonUniformityNormalized_19 | JMI; |  |  |  |  |
| F37 | Wavelet | wavelet-HHL_glszm_GrayLevelNonUniformityNormalized_47 | JMI; |  |  |  |  |
| F38 | Wavelet | wavelet-HHH_glszm_SizeZoneNonUniformity_0 | JMI; |  |  |  |  |
| F39 | Wavelet | wavelet-HHL_glszm_LowGrayLevelZoneEmphasis_47 | JMI; |  |  |  |  |
| F40 | Wavelet | wavelet-HHL_glszm_LowGrayLevelZoneEmphasis_41 | JMI; |  |  |  |  |
| F41 | Wavelet | wavelet-HHL_glszm_SizeZoneNonUniformity_2 | JMI; |  |  |  |  |
| F42 | Wavelet | wavelet-HHL_glszm_HighGrayLevelZoneEmphasis_37 | JMI; |  |  |  |  |
| F43 | Wavelet | wavelet-HHH_glszm_GrayLevelVariance_3 | JMI; MIFS; |  |  |  |  |
| F44 | Wavelet | wavelet-HHL_glszm_HighGrayLevelZoneEmphasis_34 | JMI; |  |  |  |  |
| F45 | Wavelet | wavelet-HHL_glszm_HighGrayLevelZoneEmphasis_9 | MIFS; MRMR; |  |  |  |  |
| F46 | Wavelet | wavelet-HHH_glszm_GrayLevelNonUniformityNormalized_3 | MIFS; MRMR; |  |  |  |  |
| F47 | Wavelet | wavelet-HHH_glszm_GrayLevelVariance_2 | MIFS; MRMR; |  |  |  |  |
| F48 | Wavelet | wavelet-HHH_glszm_GrayLevelNonUniformityNormalized_2 | MIFS; MRMR; |  |  |  |  |
| F49 | Wavelet | wavelet-HHL_glszm_GrayLevelVariance_46 | MIFS; MRMR; |  |  |  |  |
| F50 | Wavelet | wavelet-HHH_glszm_GrayLevelVariance_0 | MIFS; MRMR; |  |  |  |  |
| F51 | Wavelet | wavelet-HHL_glszm_GrayLevelVariance_3 | MIFS; |  |  |  |  |
| F52 | Wavelet | wavelet-HHL_glszm_GrayLevelNonUniformityNormalized_6 | MIFS; MRMR; |  |  |  |  |
| F53 | Wavelet | wavelet-HHH_glszm_HighGrayLevelZoneEmphasis_3 | MIFS; MRMR; |  |  |  |  |
| F54 | Wavelet | wavelet-HHL_glszm_GrayLevelVariance_6 | MIFS; |  |  |  |  |
| F55 | Wavelet | wavelet-HHL_glszm_GrayLevelVariance_15 | MIFS; MRMR; |  |  |  |  |
| F56 | Wavelet | wavelet-HHL_glszm_GrayLevelNonUniformityNormalized_3 | MIFS; |  |  |  |  |
| F57 | Wavelet | wavelet-HHL_glszm_GrayLevelNonUniformityNormalized_49 | MIFS; |  |  |  |  |
| F58 | Wavelet | wavelet-HHL_glszm_GrayLevelNonUniformityNormalized_36 | MIFS; |  |  |  |  |
| F59 | Wavelet | wavelet-HHH_glszm_HighGrayLevelZoneEmphasis_10 | MRMR; |  |  |  |  |
| F60 | Wavelet | wavelet-HHL_glszm_HighGrayLevelZoneEmphasis_14 | MRMR; |  |  |  |  |
| F61 | Wavelet | wavelet-HHL_glszm_HighGrayLevelZoneEmphasis_0 | MRMR; |  |  |  |  |
| F62 | Wavelet | wavelet-HHL_glszm_LowGrayLevelZoneEmphasis_9 | MRMR; |  |  |  |  |
| F63 | Wavelet | wavelet-HHH_glszm_GrayLevelNonUniformityNormalized_12 | MRMR; |  |  |  |  |
| F64 | Wavelet | wavelet-HHL_glszm_GrayLevelVariance_44 | MRMR; |  |  |  |  |
| F65 | Wavelet | log-sigma-1-0-mm-3D_firstorder_Mean_20 | Fisher; ReliefF; Alpha; FS; TS; | √ |  |  |  |
| F66 | Wavelet | log-sigma-2-0-mm-3D_firstorder_Mean_20 | Fisher; ReliefF; FS; TS; | √ |  |  |  |
| F67 | Wavelet | log-sigma-1-0-mm-3D_firstorder_Mean_21 | Fisher; ReliefF; FS; TS; | √ |  |  |  |
| F68 | Wavelet | log-sigma-2-0-mm-3D_firstorder_Mean_21 | Fisher; ReliefF; FS; TS; | √ |  |  |  |
| F69 | Wavelet | wavelet-LLH_firstorder_Variance_17 | LS; |  |  |  |  |
| F70 | Wavelet | log-sigma-2-0-mm-3D_firstorder_90Percentile_20 | LS; |  |  |  |  |
| F71 | Wavelet | wavelet-LHH_gldm_GrayLevelVariance_25 | LS; |  |  |  |  |
| F72 | Wavelet | wavelet-LHH_firstorder_RootMeanSquared_31 | LS; |  |  |  |  |
| F73 | GLCM | original_glcm_SumSquares_39 | LS; |  |  |  |  |
| F74 | First-order | original_firstorder_90Percentile_48 | LS; |  |  |  |  |
| F75 | Log-sigma | log-sigma-1-0-mm-3D_firstorder_Mean_17 | ReliefF; | √ |  |  |  |
| F76 | Log-sigma | log-sigma-2-0-mm-3D_firstorder_Mean_17 | ReliefF; | √ | √ |  |  |
| F77 | Log-sigma | log-sigma-1-0-mm-3D_firstorder_Mean_18 | ReliefF; Lasso; | √ |  |  |  |
| F78 | Log-sigma | log-sigma-2-0-mm-3D_firstorder_Mean_18 | ReliefF; | √ |  |  |  |
| F79 | Log-sigma | log-sigma-1-0-mm-3D_firstorder_Mean_19 | ReliefF; Lasso; TS; | √ |  |  |  |
| F80 | Log-sigma | log-sigma-2-0-mm-3D_firstorder_Mean_19 | ReliefF; FS; TS; | √ |  |  |  |
| F81 | Log-sigma | log-sigma-3-0-mm-3D_firstorder_Mean_19 | ReliefF; | √ | √ |  | √ |
| F82 | Wavelet | wavelet-LHL_firstorder_Mean_19 | ReliefF; | √ | √ | √ | √ |
| F83 | Log-sigma | log-sigma-1-0-mm-3D_firstorder_Skewness_0 | Alpha; | √ | √ | √ | √ |
| F84 | Wavelet | wavelet-LHH_firstorder_Median_0 | Alpha; | √ | √ | √ | √ |
| F85 | Log-sigma | log-sigma-1-0-mm-3D_firstorder_Skewness_14 | Alpha; | √ |  |  |  |
| F86 | Wavelet | wavelet-LHL_firstorder_Median_14 | Alpha; | √ | √ | √ | √ |
| F87 | Log-sigma | log-sigma-1-0-mm-3D_glrlm_LongRunEmphasis_20 | Alpha; | √ | √ | √ | √ |
| F88 | Wavelet | wavelet-LHL_firstorder_Mean_3 | Alpha; | √ | √ | √ | √ |
| F89 | Log-sigma | original_glrlm_LongRunEmphasis_33 | Alpha; | √ |  |  |  |
| F90 | Wavelet | wavelet-LLL_firstorder_Kurtosis_0 | Lasso; | √ | √ | √ |  |
| F91 | Wavelet | wavelet-LLL_glrlm_LongRunHighGrayLevelEmphasis_0 | Lasso; | √ |  |  | √ |
| F92 | Wavelet | wavelet-LLL_gldm_LargeDependenceHighGrayLevelEmphasis_0 | Lasso; | √ | √ | √ | √ |
| F93 | Wavelet | wavelet-HLL_firstorder_Minimum_16 | Lasso; | √ | √ | √ | √ |
| F94 | Wavelet | wavelet-HLL_firstorder_Median_17 | Lasso; | √ | √ | √ |  |
| F95 | Wavelet | wavelet-HHL_firstorder_Skewness_17 | Lasso; | √ | √ | √ | √ |
| F96 | Wavelet | wavelet-LHL_glcm_Imc2_18 | Lasso; | √ |  |  |  |
| F97 | Log-sigma | log-sigma-2-0-mm-3D_firstorder_Skewness_19 | Lasso; | √ |  |  |  |
| F98 | Wavelet | wavelet-HLL_firstorder_Median_19 | Lasso; | √ |  |  |  |
| F99 | First-order | original_firstorder_Energy_20 | Lasso; | √ | √ | √ | √ |
| F100 | Log-sigma | log-sigma-1-0-mm-3D_glcm_MaximumProbability_20 | Lasso; | √ | √ |  | √ |
| F101 | Wavelet | wavelet-HLL_firstorder_Skewness_21 | Lasso; | √ |  | √ | √ |
| F102 | GLCM | original_glcm_MaximumProbability_26 | Lasso; | √ | √ | √ | √ |
| F103 | First-order | wavelet-LHH_firstorder_Median_45 | Lasso; | √ | √ | √ | √ |
| F104 | Log-sigma | log-sigma-2-0-mm-3D_glszm_ZoneVariance_18 | MCFS |  |  |  |  |
| F105 | Log-sigma | log-sigma-2-0-mm-3D_glszm_LargeAreaEmphasis_18 | MCFS |  |  |  |  |
| F106 | Log-sigma | log-sigma-4-0-mm-3D_firstorder_Mean_35 | MCFS |  |  |  |  |
| F107 | Log-sigma | log-sigma-4-0-mm-3D_firstorder_Mean_36 | MCFS |  |  |  |  |
| F108 | Log-sigma | log-sigma-4-0-mm-3D_firstorder_Mean_34 | MCFS |  |  |  |  |
| F109 | Log-sigma | log-sigma-2-0-mm-3D_gldm_GrayLevelNonUniformity_17 | MCFS |  |  |  |  |
| F110 | GLCM | original_glcm_DifferenceVariance_9 | MCFS |  |  |  |  |
| F111 | GLCM | original_glcm_DifferenceVariance_10 | MCFS |  |  |  |  |
| F112 | Log-sigma | log-sigma-2-0-mm-3D_gldm_GrayLevelNonUniformity_16 | MCFS |  |  |  |  |
| F113 | Log-sigma | log-sigma-2-0-mm-3D_gldm_GrayLevelNonUniformity_18 | MCFS |  |  |  |  |
| F114 | GLCM | original_glcm_DifferenceVariance_8 | MCFS |  |  |  |  |
| F115 | Log-sigma | log-sigma-1-0-mm-3D_gldm_LargeDependenceEmphasis_12 | MCFS |  |  |  |  |
| F116 | Wavelet | wavelet-HLL_firstorder_Entropy_15 | MCFS |  |  |  |  |
| F117 | Log-sigma | log-sigma-4-0-mm-3D_firstorder_Mean_49 | MCFS |  |  |  |  |
| F118 | Log-sigma | log-sigma-1-0-mm-3D_gldm_LargeDependenceEmphasis_13 | MCFS |  |  |  |  |
| F119 | Log-sigma | log-sigma-1-0-mm-3D_glrlm_RunPercentage_12 | MCFS |  |  |  |  |
| F120 | Wavelet | wavelet-HLL_firstorder_Entropy_14 | MCFS |  |  |  |  |
| F121 | GLDM | original_gldm_LargeDependenceHighGrayLevelEmphasis_13 | MCFS |  |  |  |  |
| F122 | Wavelet | wavelet-HLL_firstorder_Entropy_16 | MCFS |  |  |  |  |
| F123 | GLDM | original_gldm_LargeDependenceHighGrayLevelEmphasis_14 | MCFS |  |  |  |  |
| F124 | Log-sigma | log-sigma-1-0-mm-3D_firstorder_Mean_22 | FS; TS; | √ |  | √ |  |
| F125 | Log-sigma | log-sigma-2-0-mm-3D_firstorder_Mean_22 | FS; TS; | √ |  |  |  |
| F126 | Log-sigma | log-sigma-3-0-mm-3D_firstorder_Mean_20 | TS; | √ |  |  | √ |
| F127 | Wavelet | wavelet-HLL_firstorder_Mean_20 | TS; | √ | √ |  | √ |
| F128 | Wavelet | wavelet-HLL_firstorder_Mean_21 | TS; | √ |  |  | √ |
